# The big three perfectionism scales: A psychometric characterization of a Chinese music student population

**DOI:** 10.1371/journal.pone.0320837

**Published:** 2025-04-04

**Authors:** Qiujian Xu, Xiaoyu Wang, Mingyi Yang, Bo Wang, Dan Yang, Meihui Li, Shilin Liu, Xiubo Ren, Yintong Liu, Siqi Liu, Minghe Song, Xiaoxuan Bao

**Affiliations:** 1 School of Arts and Design, Yanshan University, Qinhuangdao, China; 2 Music College, Catholic University of Daegu, Hayang-eup, Gyeongsan-si, Gyeongsangbuk-do, Republic of Korea; 3 Design College, Catholic University of Daegu, Hayang-eup, Gyeongsan-si, Gyeongsangbuk-do, Republic of Korea; Chiba Daigaku, Japan

## Abstract

**Background:**

Perfectionism as a multidimensional personality trait, is closely related to many important factors affecting mental, emotional, and daily life, and thus has received extensive attention from researchers around the world. The Big Three Perfectionism Scale (BTPS) is a widely recognized tool in global research, providing a comprehensive framework for assessing perfectionism through three higher-order factors and ten dimensions.

**Objectives:**

This study aims to translate and validate the BTPS in the context of music student population in mainland China, assessing its cross-cultural reliability and psychometric properties to ensure its applicability within this specific population.

**Methods:**

This study evaluated the factor structure, convergent validity, predictive validity, and overall validity of the Chinese version of the BTPS. The methodology involved translating the BTPS into Chinese and conducting cross-cultural validation through experiments with a substantial sample of musical expertise from Chinese mainland (n ≥  442).

**Results:**

The results aimed to support the hypothesis that the three-factor structural model of the BTPS demonstrates invariance within this population. Preliminary experimental results indicated that the Chinese version of the BTPS has good validity in the musical expertise population in Chinese mainland (Cronbach’s alpha coefficients of 0.86, 0.95 and 0.94 for the three dimensions, respectively), and the significance of the data (KMO >  0.6, p <  0.05) suggests that it can be further analyzed by exploratory factor analysis (EFA) and validation factor analysis (CFA).

**Conclusions:**

Our preliminary results show the feasibility of cross-cultural research on the findings of psychometric instruments from other countries, and the authors’ pioneering work is also of practical significance in enriching the psychometric instruments on perfectionism in China, which will provide a valid basis for a series of future studies on perfectionism related to music psychology.

## Introduction

### Perfectionism

Perfectionism as a personality trait is characterized by the pursuit of perfection through high performance standards and overly critical assessment of an individual’s behavior [1,2]. Perfectionism can be thought of as a multidimensional personality with both adaptive and maladaptive aspects, and clinical psychologists have shown that perfectionism is a personality trait that is associated with mental health problems and poor social outcomes, so in order to be able to fully understand the multidimensional nature of perfectionism, it is crucial to examine the relationship between the different dimensions of perfectionism and social outcomes and behaviors [3].

In a broad interdisciplinary context, perfectionism correlates with multidimensional physical and psychological manifestations. There is a high degree of correlation between perfectionism and psychopathology [4], and its dimensions are strongly associated in different ways with symptoms of obsessive-compulsive disorder, procrastination, and depression, respectively [2]. Research has shown that perfectionism is strongly associated with symptoms of low quality sleep [5], anxiety and depression [2], self-esteem [6], stress [7], and burnout [8]. In addition, perfectionism can lead to the development of psychotic disorders [4] and obsessive-compulsive disorder [9], and perfectionist students are also at risk of suffering from academic procrastination, making perfectionism one of the hot topics in psychology and psychopathology research.

Stage performance is a temporal art, usually a one-time event, and although psychologists define practice for stage performance as the enhancement of skills through repetition, stage practice requires the interaction of a variety of activities [10]. Therefore, in terms of music performance characteristics, music performance activities or music performance professional performers are characterized by perfectionism. In the literature on perfectionism as it relates to music, perfectionism is characterized by both positive and negative aspects. Research has found that the pursuit of perfectionism can be highly motivating and lead to a number of achievement-related benefits [11–13]. In contrast, perfectionist concerns have no such benefits and instead bring about more negative emotional experiences such as performance anxiety [13]. It is therefore argued that perfectionism is also a personality trait that contributes to anxiety and distress in musical performance [11,14]. In a Norwegian study, it was found that symptoms of anxiety or depression were common among musicians compared to the general population [15]. Many musicians suffer from performance anxiety during musical performances [16], in turn, the constant stress of music lessons, music practice, music playing and performance may lead to symptoms of somatic complaints and emotional fatigue in young musicians [17]. As a result, professional musicians perceive perfectionism as both central to their success and a source of problems in their professional and individual lives [18], which has led to a higher standard of perfectionism among the population practicing music professionally, which triggers the onset of music performance anxiety. Related perfectionism research in recent years has had a positive effect on the improvement of pathology in psychologically ill populations, but further evidence-based research is needed in focusing on the relevance of perfectionist performance in music professional populations and in stage performance activities.

### The Big Three Perfectionism Scale (BTPS)

**Fig 1:**
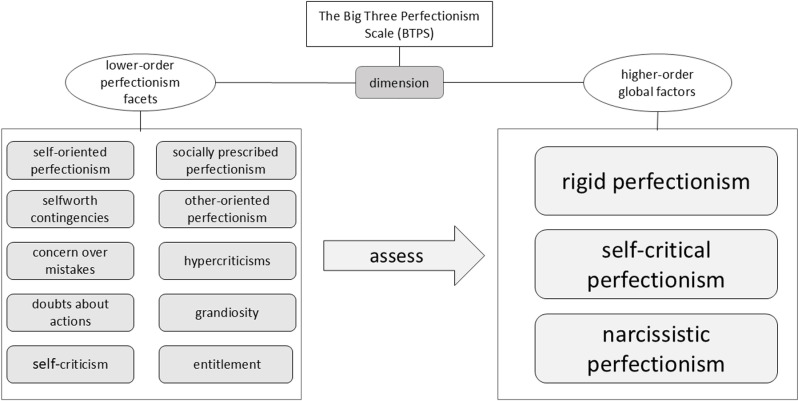
The Big Three Perfectionism Scale Dimension Divisions. This is a diagram of a theoretical model of the factor structure of the three big perfectionism scales, which contains two oval-shaped text boxes on the left and right. Below the oval text box on the left is a rectangular text box that includes ten lower-order perfectionist factors, where each gray rectangular text box represents a lower-order factor. The arrows in the middle point to the three higher-order factors of the three big perfectionisms, represented by three gray rectangular text boxes.

In 2016, Smith et al. designed a 45-item self-report questionnaire, the Big Three Perfectionism Scale (BTPS), on top of other theoretical models of perfectionism. As shown in Fig 1, the Big Three Perfectionism Scale (BTPS) first identifies 10 dimensions of perfectionism, i.e., self-oriented perfectionism [5 items], self-worth contingencies [5 items], concern over mistakes [5 items], doubts about actions [5 items], self-criticism [4 items], socially prescribed perfectionism [4 items], other-oriented perfectionism [5 items], hypercriticism [4 items], entitlement [4 items], and grandiosity [4 items], and categorized each of these 10 facets into the three higher-order dimensions of perfectionism [19]. The BTPS constructed using the taxonomy allows for a fine-grained analysis of multidimensional perfectionism, mitigates theoretical confusion, reduces the likelihood of missing core elements, and provides greater assessment reliability and precision in measuring perfectionism [20].

**Fig 2:**
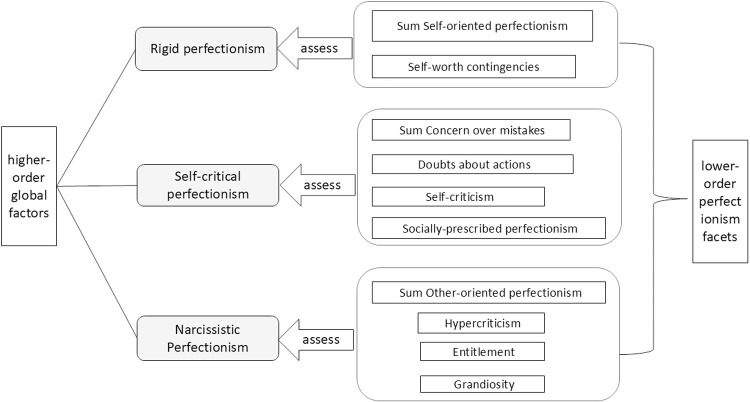
The Big Three Perfectionism Scale Dimension Divisions. This is a diagram of a theoretical model of the three big perfectionism scales, which has a curly bracket and white rectangular text box on the left and right sides representing the higher-order and lower-order perfectionism factors, respectively. In this case, the ten rectangular text boxes on the right-hand side represent each of the ten lower-order aspects of perfectionism, and they are grouped in center brackets. The arrows in the middle point to each of the three higher-order factors of perfectionism, all indicated by gray text boxes.

The BTPS categorizes perfectionism into three higher-order dimensions, namely, rigid perfectionism, self-critical perfectionism, and narcissistic perfectionism. As shown in Fig 2, the first higher-order factor of the BTPS is called “ rigid perfectionism”. This label was inspired by the subscale of the same name from the Personality Inventory for the Diagnostic and Statistical Manual of Mental Disorders-5 (PID-5) [21–23]. In contrast, the items corresponding to this dimension were written specifically to capture strict adherence to the self, i.e., one’s performance must be perfect and there is no room for error. Rigid perfectionism consists of two main lower-order aspects, self-oriented perfectionism (SOP) and self-worth contingencies (SWC). The self-Oriented Perfectionism (SOP) is the belief that it is important to strive for perfection and flawlessness [24]. The self-worth contingencies (SWC) is the tendency to base self-worth on unrealistically high standards that are often difficult to achieve [25].

The second higher-order factor of the BTPS is called “self-critical perfectionism.” Smith et al. followed the model proposed by Dunkley et al. [26], the self-critical perfectionism was divided into four lower-order aspects, namely, concern over mistakes (COM), doubts about actions (DAA), self-criticism (SC), and socially prescribed perfectionism (SPP). The concern over mistakes (COM) is the tendency to react excessively negatively to perceived mistakes and failures [2].The doubts about actions (DAA) refers to repeated uncertainty about self-performance [2]. The self-criticism (SC) is the tendency to critically criticize oneself when performance is not up to the high standards expected of oneself [26].The socially prescribed perfectionism (SPP) is when individuals subjectively believe that other people have strict and perfectly high standards for themselves as well [1].

The third higher-order factor of the BTPS, referred to as “narcissistic perfectionism,” is based on Nealis et al.‘s model [27], which categorizes it into four lower-order facets, namely, other-oriented perfectionism (OOP), hypercriticism (HC), entitlement (ENT), and grandiosity (GRAN).The other-oriented perfectionism (OOP) is the tendency to have unrealistic expectations of others [1].The hypercriticism (HC) is defined as having harsh demands on others and belittling others for imperfect performance [27].The entitlement (ENT) is the belief that one is entitled to perfect or special treatment [27].The grandiosity (GRAN) is the consistent belief that one is perfect and superior to others in comparison to others [27,28].

### The current state of cross-cultural research on the three major perfectionism scales

The three perfectionism scales have consistently demonstrated good validity in multicultural contexts and have potential for development and use in China. Since the original versions of the three major perfectionism scales are in English, regions whose native language is not English will have some resistance in the process of research and dissemination due to the language limitation. Therefore, in order to be able to use the three major perfectionism scales cross-culturally for smoother and easier perfectionism measurement, many researchers will localize them. The Persian version of the Big Three Perfectionism Scale was localized in Iran, and the research team used standard back-translation techniques to translate the scale into Persian, which is not fundamentally different from the original English version [3]. The translation process of the Turkish version of the three major perfectionism scales is not much essentially different from that of the Persian version of the scales mentioned above. Translation experts from Turkey evaluated and discussed the translation results in terms of semantic and cultural differences, and argued the validity of the Turkish versions of the three major perfectionism scales [29]. The Big Three Perfectionism Scale‐Short Form (BTPS‐SF) from Italy was developed by Di Fabio et al. [30] based on the English version of the BPTS [19]. They identified a satisfactory three-factor structure (CFI =  0.91; TLI =  0.90; Cronbach’s alphas ranged from 0.83 to 0.89), each factor contains six items, ranked on a 5-point Likert scale (from 1 =  Strongly Agree to 5 =  Strongly Disagree) [31].

For the cross-cultural study of the Chinese versions of the three major perfectionism scales, a sample of adolescents from Taiwan, China [32] and Chinese mainland [33] were selected for the study. Both studies showed good psychometric validity, but both had some group limitations in their use. In China, in-depth research on perfectionism and the development of perfectionism-related scales are not particularly extensive, and in-depth research on the three major perfectionism scales has not yet been widely conducted in Chinese mainland, so cross-cultural research on the Chinese versions of the three major perfectionism scales is necessary.

### The present research

The purpose of this study is to translate and validate the Chinese version of the Big Three Perfectionism Scale (BPTS), and it is a cross-sectional study on the psychometric properties of perfectionism for a music learning population (which includes both current college students and graduated populations) in Chinese mainland, to investigate whether the Big Three Perfectionism Scale (BTPS) can serve as a tool to measure perfectionist tendencies in a population of music majors in Chinese mainland and to propose the following two hypotheses based on the purpose of the study:

Hypothesis 1: The factor structure of the Chinese versions of the three major perfectionism scales will be reliable in the specific frame of the Chinese sample;

Hypothesis 2: The three-dimensional structural model of the three major perfectionism scales will show stability in a sample of music professional groups in Chinese mainland, satisfying a cross-cultural study of the three major perfectionism scales.

## Materials

### General Information Questionnaire (GIQ)

This is a self-administered questionnaire to collect basic information about the subjects. It consists of 4 questions that take about 2 min to complete and covers the gender, education, region and specialty of the subject. This study required subjects to be enrolled or practicing in music and music-related majors, and therefore did not require and collect the age of the subjects.

### The Big Three Perfectionism Scale (BTPS)

The BTPS is a 45-item multidimensional perfectionism self-report scale that is divided into three main higher-order dimensions, rigid perfectionism, self-critical perfectionism, and narcissistic perfectionism, designed to address perfectionism and was originally developed by Smith et al. [19]. Each item of the BTPS was rated on a 5-point Likert scale (1 =  strongly disagree, 5 =  strongly agree). All participants received the translated Chinese version of the 45-item BTPS scale.

The GIQ and the Chinese version of the BTPS will be made available to the public as separate sections and will be seamlessly integrated into a single link to facilitate distribution to a wider range of participants in the formal experiment.

## Method

### Participants and the sampling process

Convenience sampling techniques were used to recruit participants online [34], and the online questionnaire randomly distributed cross mainland China through social media (WeChat and email). Inclusion criteria were as follows: participants must be (i) native speakers of Chinese, (ii) the participants all have a bachelor’s degree or higher, the majors are music and music-related professional groups (Music and music-related professional groups: The types of music training refer to the criteria of the Chinese Ministry of Education’s *Catalogue of Undergraduate Majors in General Colleges and Universities (*普通高等学校本科专业目录), specifically: music performance and music theory. These include music performance (vocal music, instrumental music, popular music, etc.); composition and compositional technology theory (composition and electronic music and sound design); musicology (music history and theory, ethnomusicology, music culture communication, etc.); music education; music and dance studies; and music production and recording arts.) [35]. Additionally, questionnaires that meet any of the following criteria will be considered invalid during data collection and processing: (i) participants with less than a bachelor’s degree; (ii) Participants whose field of study is not music; (iii) Less than five minutes to answer questions.

Prior to the formal experiment in April 2025, 107 questionnaires were distributed for the pre-experiment (21 February 2024), with 75 sent via WeChat and 32 via email. A total of 62 valid responses were collected, yielding a 57% response rate. Table 1 presents the demographic information of the pre-experimental sample, including gender, academic qualifications, and major. These samples align with the inclusion and exclusion criteria established for the formal trial.

**Table 1:** The demographics of the pre-test participants.

Items		Number of people	Percentage (%)
Gender	Male	19	30.6%
	Female	43	69.4%
Academic Qualifications	Bachelor’s degree in progress	19	30.6%
	Master’s degree in progress	11	17.7%
	PhD currently enrolled	7	11.3%
	Bachelor’s degree	22	35.5%
	Master’s degree	2	3.2%
	Doctor’s degree	1	1.6%
Major	Music performance	37	59.7%
	Music-related majors (non-music performance)	25	40.3%

The ethics committee of Qinhuangdao First Hospital approved all aspects of this research (ID: 2023L002, through a written document). Data collection will be conducted through the local Chinese online questionnaire platform “Wenjuan.com” (Questionnaire.com). Prior to the start of the test, participants will be informed of the significance, purpose and details of this study by reading the textual content on the first page of the questionnaire. Upon reading the letter and initiating the test, participants will be deemed to have provide consent for the use of the information they provide in the test for this study. Participation is entirely voluntary, and participants retain the right to terminate the test at any time. Online participants will access the full survey via QR code scan or web link. The formal participant group will have a balanced distribution in terms of gender and region, reflecting the diversity and actual proportions of the China’s population.

### Translation

The translation procedure for the Chinese version of the BTPS followed standard back-translation procedures [36] and was reviewed by experts. Both translation and back-translation were carried out in accordance with the following steps: a. the English version of the BTPS was translated into Chinese by two bilingual master’s students, producing two different Chinese versions; b. a professional translator compared the two Chinese versions and assessed their semantic and cultural differences to form a single Chinese version; c. the first author examined the version and compared it with the original version, making suggestions and minor adjustments; d. a language expert and a translator will back-translate the Chinese version into English; e. the first author and a bilingual PhD student check and confirm the final version. The final Chinese version is not substantially different from the original English version.

### Data analysis

#### Sample profile.

Following the recommendation by Ryan (2013), the sample size for this paper was calculated using the Normal Approximation method of the PASS software (PASS 15 Power Analysis and Sample Size Software, 2017, NCSS, Kaysville, Utah, USA, ncss.com/software/pass) [37]. First, we reviewed the reliability values (Cronbach’s alpha) reported in previous studies (see Table 2), and then calculated the mean reliabilities for each of the three dimensions of the three major perfectionism scales, i.e., rigid perfectionism, self-critical perfectionism, and narcissistic perfectionism.

**Table 2:** Expected internal consistency reliability statistics (Cronbach’s Alpha) and expected sample sizes for the higher-order dimensions of the three major perfectionism scales. For comparison purposes, values from five previous reports are provided.

Item	Rigid perfectionism	Self-critical perfectionism	Narcissistic perfectionism	Sample Numbers
Smith et al. 2016 [19]	0.94	0.95	0.96	N = 288
Besharat and Atari 2017 [3]	0.88	0.94	0.94	N = 275
Kilmen and Arikan, n.d. 2019 [29]	0.89	0.90	0.90	N = 609
Wu, 2023 [32]	0.77	0.90	0.83	N = 530
Duan et al. 2019 [33]	0.83	0.88	0.86	N = 468
Average α/Assuming expected values	0.86	0.91	0.90	
Current study	N = 152	N = 442	N = 196	**N ≥ 442**

Cronbach’s alpha coefficients for the three dimensions were 0.86, 0.95, and 0.94

Next, they were used as hypothesis confidence calculated as hypothesis expectation and the relatively lower of these values [29] was used as the null hypothesis (P0) for the sample size calculation.

The results show that for the whole test, rigid perfectionism (P0 =  0.86) requires a sample size of 152 to reach 90% power (P1 =  0.94), self-critical perfectionism (P0 =  0.91) requires a sample size of 442 to reach 90% power (P1 =  0.95), and narcissistic perfectionism (P0 =  0.90) requires a sample size of 196 to achieve 90% power (P1 =  0.96). Based on the above results, we use the maximum sample size (N =  442) as the reference sample value for the Chinese version of BTPS, and the target sample size for the formal experiment will be more than or equal to 442.

#### Statistical analyses.

The Statistical Package for the Social Sciences (SPSS, version 24.0) and Amos (version 24.0) will be used for the various analyses of this study which include descriptive analysis, item analysis, exploratory factor analysis (EFA) and confirmatory factor analysis (CFA).

The Cronbach’s alpha coefficient will be used to assess the internal consistency of the BTPS scale, which has been preliminarily translated into Chinese. A value above 0.70 indicates that the scale has good internal consistency. Since the BTPS already has good dimensionality and has been validated by several cross-cultural research versions, the factor structure analysis will be conducted on the basis of the original dimensions. At this stage, the Chinese version of the BTPS scale is consistent with the original version in terms of items and dimensions. It is necessary to validate its structural validity, provided that the original alpha coefficient and KMO value (KMO >  0.6) meet the standards.

**Fig 3.**
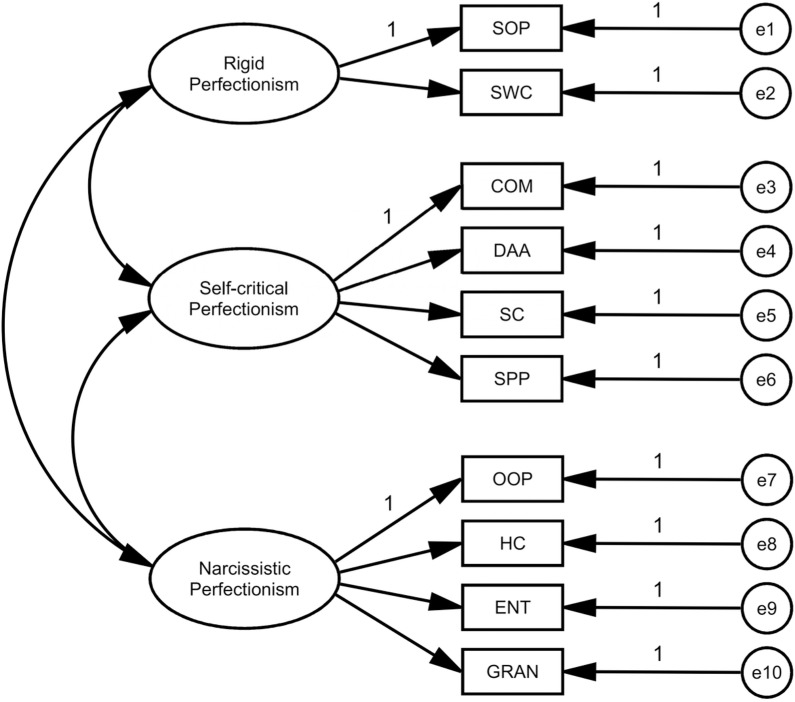
Structural Equation Modeling Diagram of the Big Three Perfectionism Scales. This is a structural equation model of the three big perfectionism scales. The three oval text boxes represent each of the three higher-order dimensions of the three major perfectionism scales, and the rectangular text boxes on the right side represent the ten aspects of the scales, with the corresponding residuals for each aspect represented by circular text boxes.

Structural equation modeling (SEM) will be used to test the prediction of the Chinese version of BTPS with the aim of guaranteeing the validity, and the structural equation modeling diagrams will be created using Amos software, see Fig 3.

The Chinese version of the scale will be validated according to the three-factor model of the original scale, and the model will be corrected several times according to the modification index (MI), after correction, the standardized loading value of the factor model is between 0.65, the residuals are positive and statistically significant, and the overall fit of the model is more satisfactory.

In this study, multiple sets of CFAs were used to test measurement invariance in the Chinese sample. Indicators of model fit included: Goodness-of-Fit Index (GFI) >  0.8 is acceptable and GFI >  0.9 is a good fit [38]; Adjusted Goodness-of-Fit Index (AGFI) >  0.9 good fit, AGFI >  0.8 acceptable [39]; Comparative Fit Index (CFI) >  0.9 good fit [38]; Tucker-Lewis Index (TLI/ NNFI) >  0.9; Root Mean Square Error of Approximation (RMSEA) <  0.08 [38]; Standardized Root Mean Square of Residuals (SRMR) <  0.08 [40]. Additionally, we will use the Ratio Between Chi-Square and Degrees of Freedom (CMID/DF) as a secondary reference indicator, with CMID/DF <  3 indicating good fit and CMID/DF <  5 considered acceptable [41]

Assuming that the overall model fit is satisfactory, this section will attempt multi-group confirmatory factor analysis (CFA) based on the original validity results and the factor structure results of the BTPS facets from 10 exploratory factor analyses (EFA). This will assess whether the refined model exhibits high stability. The convergent validity of the Chinese version of BTPS will also be an important metric for this validation. Prior to testing the measurement model, the reliability and validity of the scale needs to be tested. The reliability test was judged by looking at two indicators, Composite Reliability (CR) and Average Variance Extracted (AVE). Consistency between the two question items of the measurement scale is generally considered acceptable when CR >  0.7, AVE >  0.5 [42]. In this study, the test of convergent validity will be completed by Amos version 24.0 and the values of these two indicators will be calculated separately, assuming that the data scores CR are all greater than 0.7, AVE are all greater than 0.5, which indicates good internal consistency within the questions and acceptable reliability. The results of the multi-group CFA will also serve as potential indicators for verifying the convergent validity of the Chinese version of the BTPS scale.

To assess the stability of the test over time, 10% of the participants will be retested within a 4-week interval (refer the suggestion of Brown & Richard (2003) [43]). The reliability of the retest will be evaluated using Pearson correlation analysis. Additionally, factor analysis will be employed to assess the structural validity of the scale.

## Pre-testing and future result

Translated into Chinese according to standardized back-translation procedures. On the basis of respecting the original meaning of the scale, expressions that do not conform to the rules of Chinese expression have been adjusted, particularly in the differences in parts of speech, meanings, and sentence structures between the two languages. This is essential to ensure that the Chinese-speaking population can accurately understand the intended meaning of the original scale. For example, item 25 of the English version, “I feel uncertain about most things I do,” and item 32, “I tend to doubt whether I am doing something ‘right’,” both belong to the “Doubts about action” facet of the ten lower-order perfectionism dimensions. Consequently, their meanings are very similar when translated into Chinese. Therefore, during the translation process, item 32 emphasizes “self-doubt” more distinctly compared to item 25, which helps differentiate the degree of the concepts. Subsequently, in-depth interviews were conducted with pre-test participants through the cultural debugging program to understand their understanding of the test items. Based on the content of their feedback, the Chinese version of the scale was modified while retaining the original structure of the scale. The Chinese version of the three major perfectionism scales was used as a pre-experimental subject-completed material.

As shown in Table 2, the scale scores of the pre-experimental sample were counted and listed in advance. In addition, preliminary reliability analysis of the Chinese version of the BTPS was conducted in this study, and the results showed that the Chinese version of the BTPS presented good validity (response rate of 100%, Cronbach’s alpha coefficients for the three dimensions were 0.86, 0.95, and 0.94), and the preliminary data analysis showed signs of support for the hypotheses of this paper.

This study will first measure the original reliability and internal consistency of the Chinese version of the BTPS scale to facilitate subsequent factor analysis. Table 3 will list whether all factors of the BTPS scale, in its preliminary Chinese translation, demonstrate adequate Kaiser-Meyer-Olkin sampling adequacy measures (KMO >  0.6) and significance (p <  0.05), as well as whether Bartlett’s test of sphericity was significant for all EFAs (ps <  0.001), and whether all facets were one-dimensional. Assuming that the dataset shows significant results, this section will clarify that the resulting data is suitable for exploratory factor analysis (EFA).

**Table 3:** KMO values and cronbach’s alpha coefficients for the Chinese version of BTPS.

Factor	Cronbach’s Alpha	No. of items	KMO	Variance explained (%)
SOP		5		
SWC		5		
COM		5		
DAA		5		
SC		4		
SPP		4		
OOP		5		
HC		4		
ENT		4		
GRAN		4		
RP		10		
SP		18		
NP		17		
SUM		45		

Note. SOP =  self-oriented perfectionism; SWC =  self-worth contingencies; COM =  concern over mistakes; DAA =  doubts about action; SC =  self-criticism; SPP =  socially-prescribed perfectionism; OOP =  other-oriented perfectionism; HC =  hypercriticism; ENT =  entitlement; GRAN =  grandiosity; RP =  rigid perfectionism; SP =  self-critical perfectionism; NP =  narcissistic Perfectionism; KMO >  0.6; Cronbach’s Alpha >  0.7.

The study will perform 10 EFAs to examine the factor structure of various aspects of the Chinese version of BTPS. Tables 3 and 4 will present the means, standard deviations, and internal consistency coefficients (Cronbach’s Alphas) for each aspect of the Chinese version of the BTPS with respect to the factors and will evaluate whether all aspects within the scale and the three higher-order factors show a high degree of internal consistency with respect to each other, to further argue for bivariate correlations with the factors. In general, a Cronbach’s alpha coefficient greater than 0.9 implies that the internal consistency of the scale is very high; when the Cronbach’s alpha coefficient is between 0.7 and 0.9, it implies that the internal consistency of the scale is good; and when the Cronbach’s alpha coefficient is less than 0.7, it indicates that the degree of inconsistency of each question item in the scale is high, and the scale needs to be revised.

**Table 4:** Correlation and descriptive statistics between factors of BTPS.

	SOP	SWC	COM	DAA	SC	SPP	OOP	HC	ENT	GRAN	RP	SP	NF
SOP	1												
SWC		1											
COM			1										
DAA				1									
SC					1								
SPP						1							
OOP							1						
HC								1					
ENT									1				
GRAN										1			
RP											1		
SP												1	
NP													1
M													
SD													

Note. SOP =  self-oriented perfectionism; SWC =  self-worth contingencies; COM =  concern over mistakes; DAA =  doubts about action; SC =  self-criticism; SP P = socially-prescribed perfectionism; OOP =  other-oriented perfectionism; HC =  hypercriticism; ENT =  entitlement; GRAN =  grandiosity; RP =  rigid perfectionism; SP =  self-critical perfectionism; NP =  narcissistic Perfectionism; **p <  0.01.

The model comparison will serve as a safeguard to be able to determine the structural validity and measurement invariance of the Chinese version of the BTPS results. The model of the Chinese scale will be validated according to the three-factor model of the original scale. Model fit for the Chinese version will be reported using maximum likelihood estimation, and the model will be corrected several times according to the modification index (MI). After correction, the standardized loadings of the factor model are between 0.65, and the residuals are positive and statistically significant, so that the overall fit of the model is more satisfactory (See Table 5 in detail).

**Table 5:** Structural equation modeling fit indicators.

Indicators of judgement	Goodness-of-fit values	Reference standard value	Conclusion
χ2		the smaller the better	
df			
χ2/df		< 5 acceptable; < 3 good fit	
GFI		> 0.8 acceptable; > 0.9 good fit	
AGFI			
CFI		> 0.9 good fit	
TLI			
RMSEA		< 0.1 acceptable; < 0.08 good fit	
SRMR		< 0.08 good fit	

Note. χ2 =  chi-square; df =  degrees of freedom; GFI =  goodness-of-fit index; AGFI =  adjusted goodness-of-fit index; CFI =  comparative fit index; TLI =  Tucker-Lewis fit index; RMSEA =  root mean square error of approximation; SRMR =  standardized root mean square of residuals.

Then, we will present the dataset for multi-group confirmatory factor analysis (CFA) based on the original validity results and the factor structure results of the BTPS facets from 10 exploratory factor analyses (EFA). These analyses will serve as potential indicators for assessing the convergent validity of the Chinese version of the BTPS scale.

As shown in Table 6, the data provided in this section will be used to identify the most relevant patterns in the model with respect to the Chinese BTPS dimensions through potential factor correlations. Amos 24.0 will be used in the study and the values of the two indicators, Composite Reliability (CR) and Average Variance Extracted (AVE), will be calculated separately, assuming that the data scores CR are all greater than 0.7 and AVE are all greater than 0.5, which suggests that the scales have good internal consistency and the reliability is acceptable.

**Table 6:** Convergent validity test.

Factor	Item	Significance estimate				Title reliability		Compositional reliability	Convergent validity
		Un Std.	S.E.	z-value	P	Std.	SMC	CR	AVE
Rigid perfectionism									
Self-critical perfectionism									
Narcissistic Perfectionism									

Note. Un Std. =  Unstandardized factor loadings; Std. = Standardized factor loadings; SMC =  Std.^2^; CR =  compositional reliability; AVE =  convergent validity; CR >  0.7; AVE >  0.5; ***p <  0.01; Std. >  0.6.

Ten percent of the participants in the sample will be retested after four weeks to assess the temporal stability of the scale. The reliability of the modified Chinese version of the BTPS scale will be evaluated using Pearson correlation analysis to ensure the scale’s robustness for subsequent widespread use.

## Discussion & conclusion

A large number of music learners and performers exhibit perfectionist personality traits [12,14,17,44]. However, perfectionism is a double-edged sword: while it enhances performance efficiency and achievement in music [11–13], it also significantly increases the likelihood of negative emotions among musicians [44]. The most concerning aspect is the co-occurrence of perfectionism and music performance anxiety [45]. For instance, when musicians focus on self-evaluation or external judgments, their attention often shifts to catastrophic thoughts about their performance [11]. Some musicians with performance anxiety exhibit training rigor [44,46] and excessively high self-demands during performances, which closely resemble self-oriented perfectionism under rigid perfectionism. Thus, quantifying and assessing perfectionist traits is crucial for understanding musicians’ behavior and enhancing performance outcomes [47,48].

Chinese musical expertise populations display significant cultural heterogeneity in areas such as music processing ability [49], music perception [50,51], and musical cultural adaptation [52]. However, the absence of effective tools for quantifying perfectionist traits has significantly hindered our understanding of Chinese music practitioners. The present study aims to address this pressing issue by translating and validating the Big Three Perfectionism Scale (BTPS) in the context of Chinese culture.

Preliminary studies show promising results supporting the hypothesis. The internal consistency reliability (Cronbach’s Alpha) of the translated BTPS for the higher-order dimensions of the three major perfectionism scales did not fall below the validity shown in other cultural contexts [53,54]. Except for data related to rigid perfectionism, which aligned with the mean, all other dimensions exceeded the average significantly. We recognize that the limited sample size reduces the persuasiveness of the pilot data. Clearly, a larger sample size is necessary to further assess the applicability and validity of the BTPS. The results of formal experiments may provide valuable information regarding the factorial structure, measurement invariance, convergent validity, and predictive validity of the Chinese version of the BTPS. These findings will contribute to a deeper understanding of perfectionist traits within this population and enrich the knowledge and development of music therapists, educators, performers, and other professionals in the field.

One advantage of this study is its enhancement of the BTPS’s cross-cultural applicability, advancing our understanding of perfectionist personality traits. Additionally, the recruitment of participants from a Chinese musical cultural background offers a practical approach for Chinese researchers to explore music performance and learning behaviors from the perspective of perfectionist traits.

## Limitation

In this study, all analyses were experiments conducted on a sample of the music major study population; therefore, there may be differences in the use of the experimental results of this study to measure the psychology of perfectionism in other populations, and generalizability may easily be missing. Further cross-gender research was not conducted in this study; therefore, it is feasible to conduct cross-gender research to measure invariance for Chinese samples in future studies.
